# Metabolism of CO and H_2_ by pioneer bacteria in volcanic soils and the phyllosphere

**DOI:** 10.1093/ismejo/wraf053

**Published:** 2025-03-16

**Authors:** Nicola Fantom, Robin A Dawson, Edina Prondvai, Philippe Constant, Gary M King, Hendrik Schäfer, Marcela Hernández

**Affiliations:** School of Biological Sciences, University of East Anglia, Norwich NR4 7TJ, United Kingdom; School of Biological Sciences, University of East Anglia, Norwich NR4 7TJ, United Kingdom; School of Life Sciences, University of Warwick, Coventry CV4 7AL, United Kingdom; Centre Armand-Frappier Santé Biotechnologie, Institut national de la recherche scientifique, 531 Boulevard des Prairies, Laval, Quebec H7V 1B7, Canada; Department of Biological Sciences, Louisiana State University, Baton Rouge, LA 70803, United States; School of Life Sciences, University of Warwick, Coventry CV4 7AL, United Kingdom; School of Biological Sciences, University of East Anglia, Norwich NR4 7TJ, United Kingdom

**Keywords:** carbon monoxide, hydrogen, soil microbes, volcano, phyllosphere

## Abstract

Trace gas degradation is a widespread metabolic adaptation in microbial communities, driving chemosynthesis and providing auxiliary energy that enhances persistence during nutrient starvation. In particular, carbon monoxide and hydrogen degradation can be of crucial importance for pioneering microbial communities colonising new, oligotrophic environmental niches, such as fresh volcanic deposits or the aerial interface of the phyllosphere. After volcanic eruptions, trace gas metabolism helps pioneer colonisers to initiate soil formation in ash deposits and on recently solidified lava, a vital ecosystem service. Similarly, in the phyllosphere, bacteria colonising newly emerging leaves and shoots, and/or persisting on the oligotrophic surface of plants, also benefit from trace gas oxidation and, given the global size of this habitat, likely constitute a significant sink for these trace gases affecting atmospheric chemistry. Herein, we review the current state of knowledge surrounding microbial oxidation of carbon monoxide and hydrogen and discuss how this may contribute to niche colonisation in oligotrophic ecosystems.

## Introduction

Carbon monoxide (CO) and hydrogen (H_2_) are indirect greenhouse gases that react with hydroxyl radicals (^•^OH), which would otherwise oxidise the potent greenhouse gas methane (CH_4_). Depending on several variables, such as latitude, season, and regional pollution levels, atmospheric mixing ratios of CO and H_2_ can range from 40 ppb to over 10 ppm for CO [1] and ~500 ppb for H_2_ [2]. Anthropogenic activities are responsible for the emission of 60% of total CO to the atmosphere [3]. The remaining 40% is produced by natural sources, including a relatively small contribution from volcanoes [4], and larger contributions from oceans [5] and plants. This production occurs in plants aboveground via photoproduction from live biomass (50–200 Tg CO yr^−1^ [3, 6]) and photo/thermal production from dead biomass (~60–90 Tg CO yr^−1^ [7, 8]). Belowground production by roots contributes ~170 ± 260 Tg CO yr^−1^ [9]; however, the production budget does not distinguish between gross and net budgets because the atmospheric budget reflects the net flux, whereas the belowground budget represents gross production, as very little of the belowground production reaches the soil-atmosphere interface. The main sources of atmospheric H_2_ are fossil fuel utilization (11 ± 4 Tg H_2_ yr^−1^), biomass burning (15 ± 6 Tg H_2_ yr^−1^), nitrogen fixation (8 ± 5 Tg H_2_ yr^−1^), photochemical oxidation from methane (23 ± 8 Tg H_2_ yr^−1^), and photooxidation from volatile organic compounds (18 ± 7 Tg H_2_ yr^−1^) [10]. CO sinks include reactions with ^•^OH (~85%), diffusion into the stratosphere (~5%), and microbial consumption in soils (~10%) [3, 11]. H_2_ sinks include oxidation reaction with ^•^OH (~20%) and microbial oxidation in soil (~80%) [10].

Bacterial trace-gas oxidation occurs in various environments, including marine environments, and has been reviewed extensively in marine settings [12–14] as well as other environments, such as forest soils and the rhizosphere (*see*  Table 1 in [15] *and Fig. 4 in* [14]). Soil bacteria are estimated to consume ~250–300 Tg of CO [15, 16] and 60 Tg of H_2_ from the atmosphere per year [14]. Understanding how microbes grow in post-volcanic environments provides a powerful, tractable model to determine how soil microbial communities can recover following disturbance. Another understudied but potentially highly significant sink for CO and H_2_ is the phyllosphere microbiome as both H_2_- and CO-degrading bacteria have been found in association with plants (Table 1, Table 2) [17, 18]. The phyllosphere is functionally oligotrophic and exposes the microbiome to similar stresses as young volcanic soil systems. These include high UV radiation (especially in volcanic deposits), broad-range temperature fluctuations within a short time period, and considerable nutrient limitations.

**Table 1:** Summary of key aerobic CO-oxidising taxa and their habitats.

Taxa	Habitat (volcanic/plant)	CO-related genes	NCBI Reference Sequence	Reference
*Dictyobacter vulcani* strain W12^T^	Soils, Mt Zao Volcano, Japan	*coxL* (form 2)	NZ_BKZW00000000.1	[19]
*Thermogemmatispora carboxidivorans* strain PM5^T^	Geothermal biofilm, Kilauea Volcano, Hawaii (USA)	*coxL* (form 1)	NZ_JNIM00000000.1	[20]
*Cupriavidus ulmosensis* strain CV2^T^	Tephra soils, Calbuco Volcano, Chile	*coxL* (forms 1 and 2)	NZ_JAVCPL000000000.1	[21]
*Paraburkholderia terrae* strain COX	Tephra soils, Calbuco Volcano, Chile	*coxL* (forms 1 and 2)	NZ_JAUYZV000000000.1	[21]
*Kyrpidia spormannii* strain FAVT5	Pantelleria Island, Italy (volcanic island)	*coxL* (form 2)	GCA_902829265	[22]
*Phyllobacteriaceae* bacterium strain CO17	Phyllosphere of Hawthorn trees, UK	*coxL* (forms 1 and 2)	JAFNIO000000000	[17]
*Bradyrhizobium japonicum* strain 110*spc*4	Root nodule	*coxL* (form 2)	AH010242.2	[23]
*Labrenzia* sp strain M4	Aquatic and terrestrial plants	*coxL* (forms 1 and 2)	AY307902^*^ (form-1) AY307916^*^ (form 2)	[24]
*Stenotrophomonas* sp. strain LUP		*coxL* (form 1)	AY307920^*^	
*Labrenzia* sp. strain M8		*coxL* (form 1 and 2)	AY307903^*^ (form-1) AY307917^*^ (form-2)	
*Mesorhizobium* sp. strain NMB1		*coxL* (form 2)	AY307906^*^	
*Xanthobacter* sp. strain COX		*coxL* (form 2)	AY307911^*^	
*Paraburkholderia* sp. strain LUP		*coxL* (form 2)	AY307907^*^	

^*^ *coxL* accession numbers.

**Table 2:** Summary of key aerobic H_2_ oxidising taxa and their habitats.

Taxa	Habitat (volcanic/plant)	H_2_-related genes	NCBI Reference Sequence	Reference
*Kyrpidia spormannii* strain FAVT5	Pantelleria Island, Italy (volcanic island)	[NiFe] group 2a hydrogenases	GCA_902829265	[22]
*Methylacidimicrobium thermophilum* AP8	Pantelleria Island, Italy (volcanic island)	[NiFe] group 1b hydrogenase	LR797830	[25]
*Rhizobium meliloti* strains A1^*^ and A5^*^	Alfalfa root nodules	N.D.	N.D.	[26]
*Rhodococcus* strains A2^*^ and A3^*^				
*Pseudomonas* sp. strain A7^*^				
*Bacillus* sp. strains A8^**^ and A9^**^				
*Streptomyces speibonae* strain CS12H	*Capsella bursa-pastoris* shoot	*hhyL* (cluster-II)	AB894412^***^	[18]
*Streptomyces thermocarboxydus* strain AS13Y	*Arabidopsis thaliana* shoot	*hhyL* (cluster-II)	AB894413^***^	
*S. thermocarboxydus* strain AS22T		*hhyL* (cluster-II)	AB894414^***^	
*S. thermocarboxydus* strain OS2C	*Oryza sativa* shoot	*hhyL* (cluster-II)	AB894415^***^	
*Streptomyces scabiei* strain OR9T	*O. sativa* shoot	*hhyL* (cluster-I)	AB894417^***^	
*Streptomyces koyangensis* strain OR3C		*hhyL* (cluster-II)	AB894416^***^	
*Streptomyces thermospinosisporus* strain OR11H		*hhyL* (cluster-II)	AB894418^***^	

^*^H_2_ disappearance

^**^H_2_ production

^***^ *hhyL* accession numbers

N.D: Not determined

In this review, we focus on how trace gas oxidation may contribute to successful colonisation of newly emerging and/or oligotrophic niches, focusing on understanding the roles of CO and H_2_ oxidisers in volcanic deposits (initial soil development and maturation) and in the phyllosphere of terrestrial plants, particularly trees. The microbial communities colonising these environments provide key services, such as regulating the composition of the atmosphere through trace gas oxidation, carbon cycling, and maintaining the flow of reducing equivalents under nutrient limitation [15, 27].

## CO- and H_2_-oxidisers: phylogeny, enzymes, and habitats

Bacteria can oxidise highly variable CO concentrations. *Hydrogenophaga pseudoflava* (formerly *Pseudomonas carboxydoflava* [28]) oxidised CO from 10–50% (v/v) initial concentrations [29] down to ambient concentrations, whereas *Thermomicrobium roseum* [30] and various *Labrenzia* spp. [31] (formerly *Stappia* [32]) oxidised CO to sub-atmospheric levels from initial concentrations of 14 ppmv and 1000 ppmv, respectively. Additionally, CO-oxidising *Cupriavidus ulmosensis* CV2^T^ and *Paraburkholderia terrae* COX can oxidise CO at concentrations up to 10 000 ppmv [21]. CO-oxidation can occur as an oxygen-tolerant or true anaerobic process performed by distinct carbon monoxide dehydrogenases (CODH). Anaerobic Ni-dependent CODH, discussed in greater detail in previous reviews [33, 34], is encoded by *cooS* gene clusters in bacteria or *cdh* gene clusters in archaea [35]. These enzymes are widely distributed in forested, cultivated, and volcanic soils and sediments [36–39], and have the potential to catalyse the oxidation of CO to sub-atmospheric levels [37, 38, 40]. The aerobic Mo-Cu CODH is encoded by the genes *coxS, coxM, and coxL.* Of these, *coxL* encodes the large subunit bearing the active site of Mo-Cu CODH and is thus considered the functional marker gene for aerobic CO-oxidising bacteria [41]. Although mainly known for its aerobic activity, Mo-Cu CODH can catalyse the oxidation of CO to CO_2_ under anaerobic conditions as well as using alternative terminal electron acceptors such as nitrate [40]. Oxygen-tolerant CO oxidisers using Mo-Cu CODH (hereafter simply referred to as CODH) are divided broadly into two categories: carboxydovores and carboxydotrophs. Carboxydovores use CO only as a supplementary energy source [15], often at atmospheric levels [30, 31], supporting mixotrophic lifestyles in which trace gas oxidation fulfills maintenance energy requirements during periods of organic carbon starvation. In contrast, carboxydotrophs have typically been studied using much higher concentrations of CO [24, 42, 43] and can grow with CO as their sole source of carbon and energy, sometimes in parallel with H_2_ oxidation [24, 44]. The significance of this is that CO-oxidising microorganisms help regulate atmospheric CO levels by consuming it, thereby influencing greenhouse gas dynamics. Additionally, their co-occurrence with hydrogen oxidation suggests metabolic flexibility [30], enabling survival in low-nutrient environments [45].

Assimilation of carbon from CO oxidation is possible only via CO_2_-fixation pathways (e.g. Calvin-Benson-Bassham (CBB) cycle) that are present in carboxydotrophs but most likely absent in carboxydovores [44]. Furthermore, elevated concentrations of CO could result in inhibition of CO oxidation activity in carboxydovores with an assumed high-affinity CODH [24]. For example, carboxydovores *C. ulmosensis* CV2^T^ and *Pb. terrae* COX rapidly consumed 100 ppmv CO (within 96 hours), but *C. ulmosensis* CV2^T^ only consumed a significant quantity of CO from an initial concentration of 10 000 ppmv CO after 210 hours, while *Pb. terrae* COX did not consume a significant quantity of CO from an initial concentration of 10 000 ppmv over a 332-hour period. This suggested that CODH activity was inhibited or less effective at higher CO concentrations [21]. The current classification of CO-degrading bacteria as either carboxydovores or carboxydotrophs likely does not fully capture the metabolic role of CO degradation in bacteria and further research is needed to assess the regulation and function of this process.

Mo-Cu CODH can be sub-categorized based on CoxL amino acid motifs: form 1 (AYRCSFR) and form 2 (AYRGAGR). Form 1 is typically recognised as the definitive CO-oxidising CODH, whereas form 2 is a related Mo-Cu dehydrogenase, which may use CO as a non-preferred substrate [15, 23, 24]. Challenging the position of form 2 as a non-canonical CODH, two strains from the genus *Kyrpidia* were reported to oxidise CO despite possessing only form 2 CODH [22]; however, Cunliffe [41] found that carboxydovores from the marine *Roseobacter* clade possessing either form 2 alone or both forms of CODH could only oxidise CO when both forms were present, suggesting that form 2 CODH alone cannot perform CO oxidation. Due to contradicting findings and the limited number and diversity of strains tested for CO degradation capability, there is currently no consensus on the CO degradation potential of form 2 CODH. Experimental testing with a broader selection of bacteria possessing only form 2 CODH, combined with gene knock-out experiments on strains containing both forms 1 and 2 CODH, could help clarify the functional potential of form 2 CODH in CO oxidation.

Oxygen-tolerant [NiFe]-hydrogenase catalyses chemolithotrophic energy acquisition across diverse bacterial taxa (*see review in* [14]). Higher- and lower-affinity hydrogenases have been identified [30, 46–48], with high-affinity variants oxidising atmospheric H_2_ down to sub-ambient concentrations [48]. This provides supplementary energy to meet cellular requirements during mixotrophy and persistence, similar to carboxydovory, but can also promote anabolism by driving assimilation of CO_2_ through processes such as the Calvin-Benson-Bassham cycle [49]. The latter process is linked to moisture- and nutrient-limited environments, including Arctic and Antarctic soils [49]. H_2_ oxidation is widespread in the actinomycetes [50], and genetics experiments in the model soil-dwelling hydrogenotroph *Streptomyces avermitilis* demonstrated that H_2_ uptake was catalysed by spores, highlighting the importance of H_2_ oxidation during starvation. Deletion of hydrogenase-encoding genes drastically reduced survival of the spores [51], an effect also observed in the survival of *Mycobacterium smegmatis* mc^2^155 [52]. *M. smegmatis* possesses two [NiFe]-hydrogenases, each of which can oxidise H_2_ to sub-ambient concentrations [48], with group 2a [NiFe]-hydrogenase Huc expressed at the initial onset of stationary phase to promote mixotrophy, whereas group 1 h [NiFe]-hydrogenase Hhy activity was observed during long-term persistence [53], demonstrating the ability of soil bacteria to perform metabolic switches to effectively use trace gases during starvation. Similarly, *T. roseum* substantially changed its metabolism during the shift to stationary phase to take advantage of trace gases such as H_2_ and CO [30].

Genetics- and activity-based studies have indicated the global significance of CO- and H_2_-oxidisers in low-nutrient ecosystems, including marine ecosystems [54], Antarctic deserts [55, 56], and non-polar deserts such as those near hot springs in Chile [57], biological soil crusts in Israel [58], and Australian deserts [59]. Additionally, the ability to use both CO and H_2_ has been observed in bacteria from many clades [24, 30, 44, 48, 60–63]. This widespread distribution suggests that both CO and H_2_ oxidation play a crucial role in niche colonisation [20, 30, 54, 64, 65].

## Adaptations for colonising extreme environments

Oligotrophic conditions, such as those found in fresh and pristine volcanic deposits or on the surface of newly emerging plants and their compartments, pose significant challenges to many microbial processes. Some organic carbon is provided to these environments via aeolian deposition and precipitation, supporting some heterotrophic activity, but trace gas metabolism has been shown to be a key and consistent source of carbon and energy in early soil formation, particularly preceding colonisation by plants [66, 67].

The regulation of trace gas oxidation in isolated bacteria provides much insight into the conditions driving environmental fluxes. Studies have demonstrated that, in some isolates and soil microcosms, CO and H_2_ oxidation occur during organic carbon starvation, with the presence of organic carbon often repressing trace gas metabolism [16, 30, 31, 48, 61, 68, 69]. The significance of trace gas oxidation to oligotrophic ecosystems was clearly indicated when King [66] demonstrated that up to 25% of reducing equivalent flow in recent and developing volcanic deposits were due to CO and H_2_ oxidation, which may be sufficient to meet the maintenance requirements for long-term survival of cells. In another study, *M. smegmatis* was shown to use H_2_ and CO to meet its energy requirements under hypoxic and carbon limited conditions [61]; however, this is not always the case as the carboxydotroph *Mycobacterium* sp. JC1 grows mixotrophically using both CO and organic carbon with no inhibition of either process by either substrate [43], demonstrating that starvation is not the only condition that triggers CO-oxidation in bacteria. Similarly, *H. pseudoflava*, a carboxydotroph capable of oxidising ambient levels of CO [29], oxidised both CO and H_2_ whilst growing heterotrophically to drive greater assimilation of the organic substrate. The mixotrophic use of CO/H_2_ and organic carbon [65, 70], or the alternative use of CO/H_2_ as supplementary energy sources to support survival in the absence of organic carbon [30, 48, 50, 56, 61], offers significant advantages for bacteria colonising oligotrophic ecosystems. This extends to other environments, as *Armatimonadota* MAGs retrieved from marine sediments contain a NiFe group 4b hydrogenase, which is associated with CODHs, highlighting their potential ecological roles in carbon cycling [71]. King and Weber [67] postulated that CO and H_2_ oxidation are selected for during soil succession to meet microbial maintenance requirements, and Bay et al. [45] found that trace gas oxidation exceeded the minimum requirements for population maintenance in energy-limited soils.

## Trace gas oxidisers in volcanic soils

**Figure 1:**
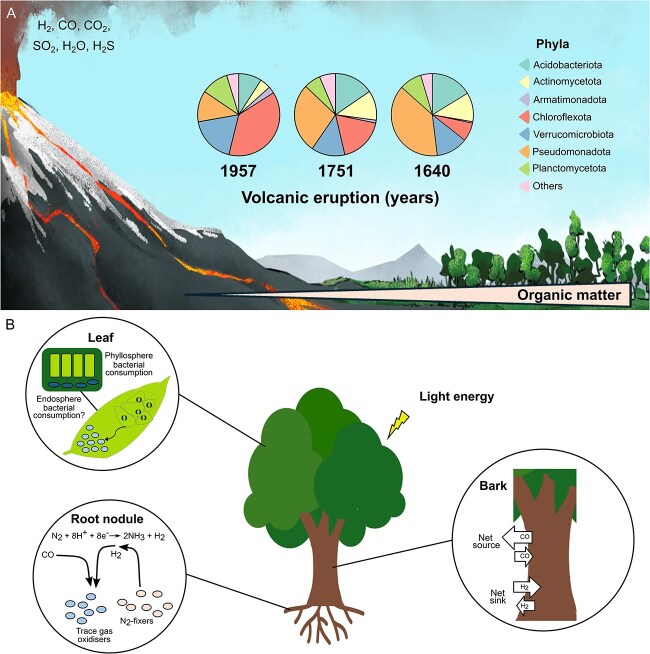
Microbial succession and carbon cycling in volcanic and plant ecosystems. A. A chronosequence approach to potential trace gas oxidisers in pioneer and established soil ecosystems, using a volcanic land formation model. Bacterial taxonomy (pie charts: 16S rRNA gene abundance) was adapted from [76]. B. Trace gas cycling in plant ecosystems, divided between rhizosphere (e.g. root nodules) and phyllosphere (above ground parts, e.g. leaves and bark). Trace gas flux from the bark ecosystem was summarized from ([92]). Numbers below the pie charts represent the year of the eruption at Llaima volcano.

The use of trace gases is thought to be a driver of microbial community development [66], a key aspect of re-establishing a thriving ecosystem following a volcanic eruption. After lava solidifies, early microbial colonisers use light and/or chemical energy (e.g. H_2_, CO, and CH_4_) to persist (Fig. 1A) [67, 72]. Some autotrophs, including chemolithoautotrophs, can utilise CO as readily available energy sources, supporting the assimilation of CO_2_ into organic carbon, which in turn can support heterotrophs and more complex life. King [66] has predicted minor net contributions of CO and H_2_ to CO_2_ fixation on unvegetated Hawaiian volcanic deposits, with H_2_ contributing substantially more than CO and rivalling inputs from wet deposition of organic matter, but highlighted the importance of trace gas oxidation to energy acquisition in organic carbon-limited sites. Many trace gas-oxidising microorganisms are also heterotrophs [20, 21, 29, 30, 73], likely facilitating growth using transient carbon depositions whereas long-term persistence is supported by trace gases, as observed in axenic cultures [16, 48, 53]. Evidence suggests that trace gas metabolism is fundamental to maintenance of healthy mature soils [27, 74] and CO oxidation is an important and widespread property of soil bacteria with CODH form 1 *coxL* genes identified in several genera belonging to seven phyla that dominate soil environments (*Pseudomonadota, Actinomycetota, Acidobacteriota, Chloroflexota, Bacillota, Gemmatimonadota, and Bacteroidota)* [16]. Some CO oxidisers containing form-1 *coxL* genes, isolated from volcanic and phyllosphere environments include *Dictyobacter vulcani* strain W12^T^, *Thermogemmatispora carboxidivorans* strain PM5^T^, *Cupriavidus ulmosensis* strain CV2^T^, *Paraburkholderia terrae* strain COX, Phyllobacteriaceae bacterium strain CO17, *“Labrenzia carboxidovorans* strain M4”, *Stenotrophomonas* sp. strain LUP, and *Labrenzia* sp. strain M8 (Table 1).

When soils and plant life are established, trace gas oxidisers remain abundant and continue to play a significant role in trace gas oxidation [67]. Studies of lava flows at different successional stages, with or without vegetation, indicate that *in situ* CO uptake rates are highest in recent, bare deposits [67]. Additionally, CO and H_2_ contribute more significantly to community respiration in these environments compared to mature, vegetated sites, where organic matter—likely provided through root exudates—is more prevalent [66, 67]. This demonstrates the impact of trace gas oxidation in nutrient-depleted environments. King and Weber [67] demonstrated that, within sites of the same depositional age, vegetated areas had a higher uptake capacity for atmospheric CO compared to bare sites, which reflects the interplay between biotic and abiotic factors driving CO production and consumption *in situ.* Factors to consider that may influence observed flux include, but are not limited to, higher *in situ* CO production, a greater abundance of CO oxidisers *in situ*, and the availability of organic matter. Trace gas oxidising microorganisms may continue to play important roles in vegetated soils through plant-microbe interactions, as CO oxidisers may contribute to N_2_ fixation on vegetated volcanic cinders [66, 67], supporting plant succession. Following plant development, roots emit CO, which can be consumed by root-associated microorganisms [9, 67] such as the CO-oxidising, nitrogen-fixing endophyte *Bradyrhizobium japonicum* [23] and several other CO oxidisers, including two nitrate-respiring isolates, *Paraburkholderia xenovorans* LB400 (formerly *Burkholderia xenovorans* LB400 [75]) and *Xanthobacter* sp. str. COX, as well as two denitrifying isolates, *Bradyrhizobium* sp. str. CPP and *Labrenzia aggregata* IAM 12614 [40]). Additionally, anaerobic CO oxidation was also widespread in lava flows of different ages, occurring under mesophilic (25°C) and thermophilic (60°C) conditions between 10 ppm and 25% (v/v) [38]. Furthermore, while H_2_ is present only as trace gas in the atmosphere, those possessing high affinity hydrogenases can oxidise it to sustain metabolism under energy-limited conditions. This is especially relevant in young volcanic deposits, where extreme oligotrophy, mineralogical constraints, and fluctuating environmental conditions create challenges for microbial survival, making trace gas oxidation a crucial metabolic strategy (Table 2).

The phylum *Chloroflexota* are of particular interest for trace gas metabolism in volcanic ecosystems due to its global prevalence [62, 76–80], dominance in recent, bare deposits [62, 76, 81, 82], and the isolation of CO- and H_2_-oxidising members from volcanic and geothermal environments (Table 1) [20, 73, 83]. *Chloroflexota* colonise diverse oligotrophic niches alongside other putative CO- and H_2_-oxidisers [84–87]. Previously, we examined bacterial diversity along a chrono-sequence path (i.e. soils of different ages) on Llaima Volcano, Chile, and found that a specific order of *Chloroflexota*, *Ktedonobacterales*, dominated the young (1957) soil [76]. Very little is known about Ktedonobacterales but our metagenomic analysis revealed that some metagenome-assembled genomes contain genes encoding CODH and hydrogenases [62]. Islam et al. [30] demonstrated the ability of two members of the *Chloroflexota* (including one member of the class Ktedonobacteria) to persist during starvation by oxidising atmospheric levels of H_2_ and CO. Further studies on chemolithoautrophy in isolates from this phylum may provide valuable insight into the colonisation of oligotrophic niches by pioneering *Bacteria*, as well as how their activity, distribution, and ecological role vary across soil carbon content gradients. This can enhance our understanding of the advantages provided by trace gas metabolism under nutrient stress conditions experienced *in situ*.

## Trace gas oxidisers in plant microbiomes

Despite the atmospheric and plant-derived sources of carbon and energy, colonisers of the aerial interface of the phyllosphere are generally exposed to oligotrophic conditions combined with fluctuating temperature, limited water availability and UV light (Fig. 1B) [88]. Still, phyllosphere communities are taxonomically and functionally diverse [89]. Photoproduction of CO and H_2_ by plants is well-known [90, 91] and largely linked to the leaf internal compartment with emission from the stomata [7]. The notion that this may provide an important and extensive niche for CO-degrading bacteria is supported by the estimate that ~25% of phyllosphere bacteria possess *cox* genes, and by the experimental finding that bacteria filtered from leaf washes consume CO [17]. For comparison, in soil microbial communities, the fraction of microorganisms able to consume H_2_ and CO was estimated to make up an average of 39% and 56%, respectively, based on short read sequence assemblies, and 26 to 31% based on metagenome assembled genomes [45]. Additionally, for marine environments, it has been estimated that ~2% of bacteria oxidise H_2_, whereas 21% oxidise CO [14]. Further evidence is provided by the *Phyllobacteriaceae* strain CO17, a CO oxidiser isolated from leaf washes, which possesses both forms of CODH, RuBisCO, and a hydrogenase linked to hydrogenogenic fermentation [17]. Other plant-associated processes support trace gas-oxidising bacteria, as N_2_-fixation generates ~9 Tg yr^−1^ H_2_ (Fig. 1B) [14] which may then be oxidised by rhizobia, endophytes, or epiphytes. Work by Kanno et al. [18] demonstrated that H_2_-oxidising bacteria with high affinity [Ni-Fe] hydrogenases colonise various plant species and can take up H_2_ at ambient levels. Their findings suggest that plant-associated H_2_ oxidation could represent a significant sink for atmospheric H_2_. Further studies are required to evaluate the significance of vegetation and phyllosphere-associated bacteria in global CO and H_2_ cycling. Estimates of global leaf surface area of terrestrial vegetation vary, ranging from satellite-based measurements of 2 × 10^8^ km^2^ to ground-based estimates of 6.4 × 10^8^ km^2^ [93]. With average bacterial densities of 10^6^–10^7^ per cm^2^, the terrestrial leaf habitat alone could host up to 10^26^ cells [89, 93]. These estimates focus on leaf epiphytic bacteria and thus provide a conservative estimate of the global aboveground plant-associated microbial population, excluding those associated with shoots, stems, and woody surfaces. Compared to soil bacteria (with an estimated global population 3 × 10^29^ cells [94]), the phyllosphere population may be small; however, unlike soil microorganisms, those in the phyllosphere are in direct contact with the atmosphere, and their role and significance in trace gas cycling require further study. Additionally, very few tree species have been surveyed for CO oxidation to date [17, 92]. As alluded to above, analyses need to be expanded to investigate the potential role of bark-dwelling bacteria and assess fluxes at ambient CO levels, particularly since H_2_-oxidising and CO-oxidising bacteria are abundant and active members of bark microbiomes [92]. Studying the phyllosphere of other species, including vascular and non-vascular plants would provide valuable insight into the ecosystem-wide dynamics of trace gas oxidation, as soils and marine CO oxidation have long been the focus. Furthermore, understanding how deforestation, changes in land management, and wildfires might influence future atmospheric CO levels is crucial.

## Challenges and future directions

Microbial trace gas metabolism is a significant activity of microbial communities with global impacts relating to soil formation, pollutant degradation, and climate regulation. The role of trace gases in driving microbial colonisation of newly emerging, temporary, and oligotrophic niches, such as fresh volcanic deposits or the phyllosphere of developing and established vegetation displaying changes in leaf and shoot phenology, is yet to be explored in greater depths by interdisciplinary approaches, including microbial ecology, biogeochemistry, and molecular biology. The limited work done so far indicates that these environments are rich in CO- and H_2_-oxidising bacteria, marking these ecosystems as ones with great and unexplored potential to understand their ecological roles in soil formation and plant resistance to biotic and abiotic stress. Additionally, a more detailed understanding of the role of plants and CO-oxidising bacteria is needed to determine how trace gas metabolism may offer competitive advantages to the pioneering bacteria which initially colonise these habitats, potentially by providing them with energy and driving chemosynthesis. Improving the understanding of regulation of trace gas-degrading organisms and enzymes will be critical for modelling the impact of environmental degradation on CO and H_2_ concentrations in the atmosphere. It may also enhance the prospects of their targeted application in mitigation of air pollution and greenhouse gases.

## Data Availability

No data were used for the research described in the article.
